# Association between dietary branched-chain amino acids and multiple chronic conditions among older adults in Chinese communities

**DOI:** 10.1186/s12986-024-00825-9

**Published:** 2024-07-30

**Authors:** Yuanfeng Song, Ji Zhang, Ziqiang Luo, Lanlan Wu, Zhaopei Cai, Xiaoqi Zhong, Xiaoxue Zeng, Tingxi Cao, Hong-en Chen, Shan Xu, Chang-yi Wang

**Affiliations:** 1 Department of Public Health and Preventive Medicine, School of Public Health, Zunyi Medical University, Zunyi, 563000 China; 2 Department of Non-communicable Disease Prevention and Control, Shenzhen Nanshan Center for Chronic Disease Control, Shenzhen, Guangdong 518000 China

**Keywords:** Isoleucine, Leucine, Branched-chain amino acids, Older adults, Multiple chronic conditions

## Abstract

**Background:**

The association of BCAAs (isoleucine, leucine, and valine) with cardiovascular and cerebrovascular diseases has been widely recognized by researchers, but there is limited evidence to support the relationship between BCAAs and multiple chronic conditions (MCCs) in older adults. This study aimed to explore the correlation between BCAA levels in the diets of older adults and MCCs.

**Methods:**

Based on a health management cohort project in Nanshan District of Shenzhen, 4278 individuals over 65 years old were selected as participants via multi-stage stratified sampling from May 2018 to December 2019. Data were collected using a validated semi-quantitative food frequency questionnaire, as well as anthropometric and chronic disease reports. MCC was defined as the coexistence of two or more chronic diseases, namely, hypertension, dyslipidemia, diabetes, CAD, stroke, CKD, and CLD. Multivariate unconditional logistic regression analysis was used to analyze the relationship between dietary BCAAs and MCCs in older adults, and then, gender stratification analysis was performed. A restricted cubic spline model (a fitted smooth curve) was used to determine the dose–response relationship of isoleucine with MCCs.

**Results:**

A total of 4278 older adults aged 65 and above were included in this study, with an average age of 72.73 ± 5.49 years. The cohort included 1861 males (43.50%). Regardless of whether confounding factors were corrected, isoleucine was a risk factor for MCCs (OR = 3.388, 95%CI:1.415,8.109). After gender stratification, the relationships between dietary isoleucine and MCCs (OR = 6.902, 95%CI:1.875,25.402) and between leucine (OR = 0.506,95%CI:0.309,0.830) and MCCs were significant in women, but not in men. No significant association between valine and MCCs was observed. In addition, isoleucine was a risk factor for MCCs when its intake was greater than 4.297 g/d.

**Conclusion:**

Isoleucine may play an important role in regulating age-related diseases. BCAAs such as isoleucine can be used as risk markers for MCCs in older adults.

**Supplementary Information:**

The online version contains supplementary material available at 10.1186/s12986-024-00825-9.

## Introduction

As the global population aging trend intensifies, the health problems of older adults are particularly important [1]. “The U.S. Department of Health and Human Services (HHS) defines multiple chronic conditions (MCCs) as having two or more chronic diseases [2–5]. More than 80% of older adults in the United States have at least one chronic disease, and the prevalence of MCCs is about 15–43% [6, 7]. A survey of 162,464 Guangdong residents in China reported that the incidence of MCCs among people over 65 years old was 47.5% [8]. Similarly, a nationally representative study showed that the probability of MCCs among older adults was 42.4% [9]. Compared to having a single chronic disease, multiple morbidity leads to a decline in quality of life, an increased risk of adverse drug events and death, and increased consumption of medical resources, which bring many challenges to the prevention, control, and management of chronic diseases.

In the process of exploring the health problems of older adults, increasing attention has been paid to the role of branched-chain amino acids. Branched-chain amino acids (BCAAs), including leucine, isoleucine, and valine, are essential amino acids for the human body, accounting for about 40% of the total amino acid requirements. BCAAs play important roles in protein metabolism and cell growth, but they cannot be automatically generated in the human body and need to be taken from external sources [10, 11]. The relationship between BCAA levels and chronic diseases, such as hypertension [12], type 2 diabetes [13], and stroke [14], has attracted the attention of researchers. However, the current research on the relationship between BCAAs and MCCs is still limited and may be affected by sample size and geographical constraints. For example, a Spanish cohort study of 1488 older people found that abnormal levels of BCAAs might be associated with an increased risk of chronic disease comorbidity, and isoleucine and valine were significantly associated with higher multi-disease scores. The levels of these BCAAs can be used as a risk marker for multimorbidity in older adults [15], who are at high risk. A cross-sectional study of 700 people aged ≥ 65 years in Spain found that the ‘amino acid/glycolysis/ketogenic’ factor was associated with a higher risk of cardiovascular metabolic comorbidities [16].

Currently, there is little evidence on the association between BCAAs and MCCs, and the findings of most studies are affected by a small sample size and regional constraints. Therefore, this study intended to explore the relationship between dietary BCAAs and MCCs (hypertension, dyslipidemia, diabetes, stroke, coronary artery disease (CAD), chronic kidney disease (CKD), chronic liver disease (CLD)) among older adults in Chinese communities. Through this study, we hope to gain a deeper understanding of the relationship between health and branched-chain amino acid metabolism in older adults, and provide new perspectives and strategies for the prevention and control of MCCs. Understanding and clarifying the relationship between dietary BCAAs and MCCs is of great significance for reducing the incidence of chronic diseases and improving the overall health status of older adults.

## Materials and methods

### Participants

The data used in this study were from a population cohort of older adults in Nanshan District. According to the national health policy of China, a cross-sectional study was conducted as part of a free health examination project for older residents in the community. The purpose was to investigate the nutrition and health status of adults aged 65 and above in China. Data were collected via stratified cluster random sampling from 53 community health service centers in 8 blocks of Nanshan District, Shenzhen City, China, from May 2018 to December 2019. Initially, a total of 4478 older people were recruited at baseline, but we developed inclusion and exclusion criteria to include only eligible participants who (i) were aged 65 or above; (ii) lived in Shenzhen for at least 6 months; (iii) had undergone annual physical examinations at community health service centers; and (iiii) voluntarily participated, agreed to complete the survey, and signed an informed consent. Participants who met any of the following conditions were excluded: (i) under 65 years of age (*n* = 3); (ii) refusal to complete the questionnaire survey (*n* = 6); and (iii) those lacked complete dietary records, those without records of their history of disease and reported implausibly low or high dietary energy intake (< 600 kcal/day or > 4000 kcal/day)(*n* = 191). Participants were given a copy of the written informed consent. The study protocol was approved by the Ethics Committee of Shenzhen Nanshan District Chronic Disease Prevention and Control Center (No. 1120180009).

Based on the inclusion and exclusion criteria, a total of 4278 eligible participants were included in the analysis.

### Dietary assessment

This study used a validated food frequency questionnaire (FFQ) [17] to assess habitual dietary consumption, which was based on food intake in the month before the interview. Due to differences in eating habits, some infrequently eaten foods were not included. A total of 62 food items were listed in the semi-quantitative 81-item FFQ, which had previously been validated using six 3-day energy-adjusted diet records of 26 nutrients among Guangzhou women [17]. Each food item had a common unit or portion size (bowl, box, cup, gram, etc.), and participants were asked to report their average food consumption at four frequencies (never, monthly, weekly, and daily). Color images of the corresponding portion of these food items were provided to help quantify the portion of food. The consumption of each food item was converted into daily intake (g/d), and dietary energy, carbohydrate, and BCAA intakes were calculated by combining dietary data with Chinese food composition tables [18, 19]. The intake of total branched-chain amino acids was calculated as the cumulative sum of three amino acids (leucine, isoleucine, and valine).

### Data collection

Data collection was mainly carried out through the use of a questionnaire, physical measurements, and laboratory tests. Each community health service center set up a survey team composed of three members. The questionnaire survey was conducted through one-on-one, face-to-face interviews with community medical staff, using a flat-panel visual questionnaire survey system with real-time recording and intelligent logic verification. Before completing the questionnaire survey, professionally trained nursing staff read the informed consent form to the participants and explained the purpose of the study, specific survey items, possible risk factors, etc. The participants then voluntarily signed the informed consent form.

1. Questionnaire survey: main demographic characteristics (gender, age, education level, marital status, body mass index(BMI), etc.), behavioral lifestyle (physical exercise, sleep status, smoking, drinking, regular night shift when young, etc.), common important diseases (hypertension, diabetes, coronary heart disease, stroke, dyslipidemia, etc.), and frequency of intake of various foods in the past month.
2. Physical measurement: weight, height, waist circumference, and blood pressure
3. Laboratory examination: Blood samples were collected as fasting venous blood in the early morning, with the participants having fasted for at least 8 h before sampling. The blood samples were detected using an automatic biochemical analysis instrument (HITACH 7080). Biomarkers of lipid metabolism, such as total cholesterol (TC), triglycerides (TG), high-density lipoprotein cholesterol (HDL-C), and low-density lipoprotein cholesterol (LDL-C), were measured.

### Outcome indicators and related definitions

The main outcome was the coexistence of MCCs. According to the risk and distribution characteristics of chronic diseases in the older adult population in China [20, 21], we focused on seven major non-communicable diseases: hypertension, dyslipidemia, diabetes, CAD, stroke, CKD, and CLD.These diseases are all over the community and of the hospital diagnosis is or suffered from these diseases.By asking the participants and inquiring about the past medical history.

Hypertension: According to the ‘Chinese Guidelines for the Prevention and Treatment of Hypertension (2018 Revision)’ [22], the diagnostic criteria for hypertension are systolic blood pressure ≥ 140 mmHg and/or diastolic blood pressure ≥ 90 mmHg. Blood pressure was measured three times on different days.“Individuals participating in the survey who were on antihypertensive medication are classified as hypertensive, regardless of whether their blood pressure readings fall below the threshold of 140/90 mmHg.

Diabetes [23]: According to the diagnostic criteria of the Diabetes Branch of the Chinese Medical Association, diabetes is diagnosed when the fasting blood glucose level is ≥ 7.0mmol/L; the random blood glucose level is ≥ 11.1mmol/L and is accompanied by obvious diabetes symptoms; the 2-hour blood glucose level of the glucose tolerance test is ≥ 11.1mmol/L; or the glycated hemoglobin level is ≥ 6.5%.Participants in the survey who are actively undergoing medication therapy for diabetes.

Dyslipidemia [24]: The diagnosis of dyslipidemia needs to meet one of the following four criteria: TG ≥ 2.3mmol/L indicates hypercholesterolemia; TC ≥ 6.2mmol/L indicates hypertriglyceridemia; HDL-C < 1.0mmol/L indicates low HDL-C; and LDL-C ≥ 4.1mmol/L indicates high LDL-C. Survey subjects who are currently undergoing pharmacological treatment for the reduction of blood lipids.

CVD: According to the definition of the American Heart Association [25], coronary blood vessels are blood vessels that provide blood to the heart. When coronary blood vessels become narrow or blocked because of atherosclerosis, myocardial ischemia, hypoxia, or necrosis, and uncomfortable feelings such as chest pain and pressure occur, leading to coronary vascular disease, including angina pectoris, myocardial infarction, and ischemic heart failure.

Stroke: The diagnosis of stroke was based on the relevant diagnostic criteria in the ‘Chinese Guidelines for the Diagnosis and Treatment of Acute Ischemic Stroke 2018’ [26] and the ‘Chinese Guidelines for the Diagnosis and Treatment of Cerebral Hemorrhage (2019) ‘ [27]. Stroke involves acute symptoms of a focal brain injury that have lasted for 24 hours or more (or lead to death before 24 hours) and are confirmed by a head CT or an MRI examination, including ischemic and hemorrhagic stroke.

CKD [28]: CKD is a progressive disease characterized by renal function proliferation and irreversible decline. It includes kidney stones, diabetic nephropathy, renal cysts, and chronic nephritis, including chronic renal insufficiency, diabetes, kidney disease, renal cysts, renal failure, kidney failure, etc.

CLD [29]: CLD represents a significant global health issue, impacting a vast demographic segment across the globe. It includes fatty liver disease and liver cyst, among others, including fatty liver, liver cyst, liver cirrhosis, etc.

Body mass index (BMI) [30, 31]: BMI is a commonly used index to evaluate health status and obesity. It is a numerical value obtained by dividing weight (kg) by the square of height (m). The formula is as follows: BMI = weight / height squared (kg/m2).

Physical activity [32]: The International Physical Activity Questionnaire (IPAQ) calculates the level of physical activity at a certain intensity per week: the MET assignment corresponds to physical activity × weekly frequency (d/w) × daily time (min/d), and divides physical activity into three levels: low-, medium-, and high-intensity exercise.

Abdominal obesity: It is defined as waist circumference (WC) ≥ 90 cm for males or ≥ 85 for females.

Smoking status: Smoking refers to the continuous or cumulative smoking of 100 cigarettes or more.

Drinking status: This refers to continuous drinking at least once a week for 6 months, with the amount being more than 50 g each time.

### Statistical analysis

An Epidata database was used for data management, and variables with less than 5% data missing were interpolated using a multiple-interpolation method. The SPSS 26.0 software was used for data analysis. If the two-tailed *P* < 0.05, the difference was considered statistically significant.

Quantitative data that did not follow a normal distribution were described using median (interquartile range). Wilcoxon rank-sum test was used for comparison between two groups, and Kruskal–Wallis rank-sum test was used for comparison between multiple groups. Qualitative data were described using the frequency or composition ratio (%), and the χ² test was used for inter-group comparison. Dietary intake of branched-chain amino acids was divided into quintiles (Q1-Q5). A restricted cubic spline model (a fitted smooth curve) was used to determine the dose–response relationship of isoleucine with MCCs.The receiver operating characteristic (ROC) curve and the area under the curve (AUC) were performed to assess the diagnostic value of isoleucine for the detection of MCCs and differentiation between grades.

Firstly, differences in the characteristics of the participants were explored according to the coexistence of MCCs and the BCAA quintiles. Then, multivariate unconditional logistic analysis was used to analyze the association between three amino acids (isoleucine, leucine, and valine) and MCCs. Model 1 did not adjust for any confounding factors. Model 2 adjusted for gender and age. Model 3 further adjusted for smoking and drinking status, BMI, marital status, exercise intensity, and educational level.

## Results

### Basic characteristics of the participants

**Fig. 1:**
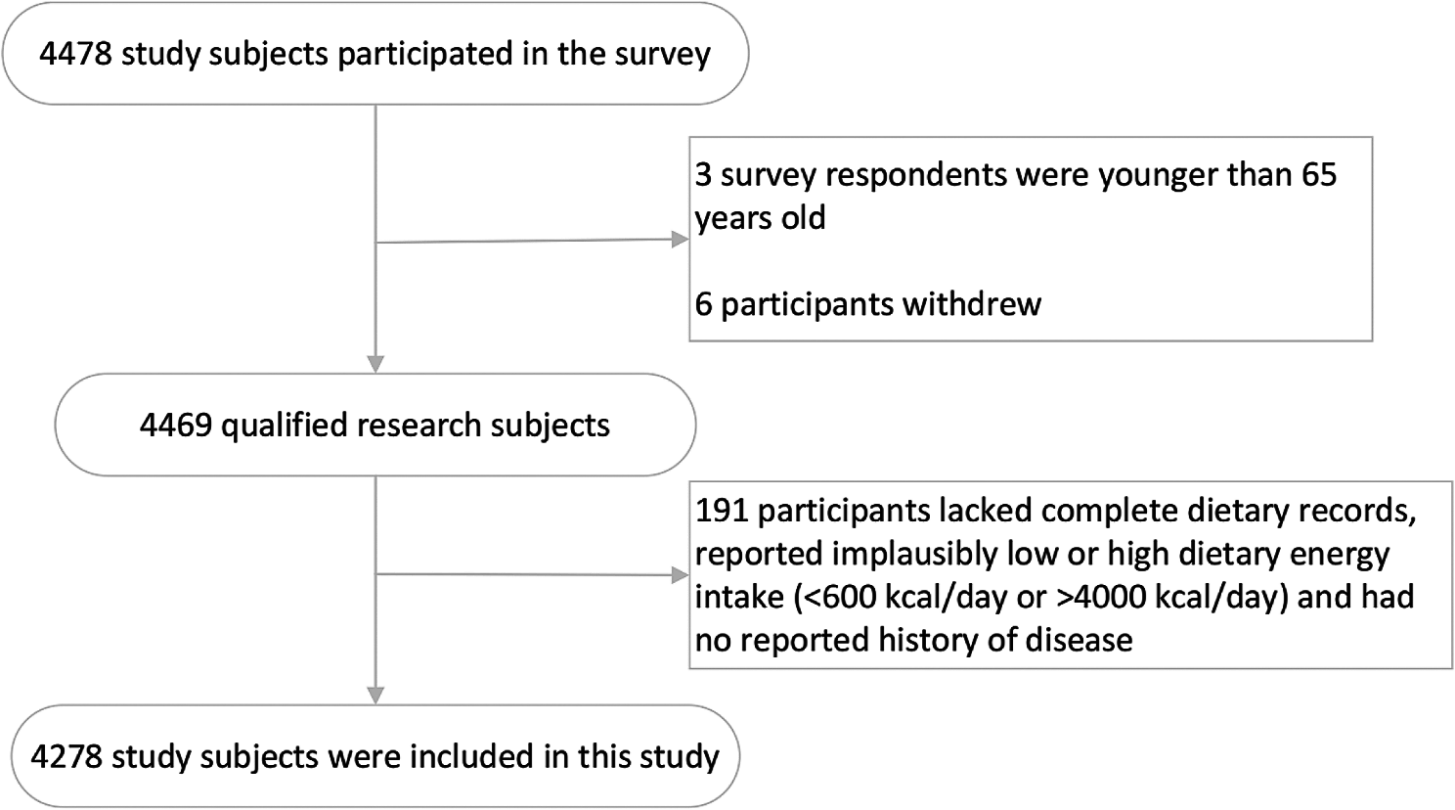
A flow chart of the participant recruitment process

A flow chart of the participant recruitment process is shown in Fig. 1. The list of English acronyms is included in Supplementary Tables 1, and the basic characteristics of the participants are shown in Table 1. Among the 4278 participants, 1861 (43.50%) were male, with an average age of 72.73 ± 5.49 years. There were significant differences between men and women in terms of age, marital status, educational level, regular night shift, smoking, drinking, sleep time, and central obesity, dyslipidemia, stroke, and CKD (*P* < 0.05). There were no significant differences between men and women in terms of household registration, exercise intensity, BMI, BP, TC, TG, HDL, and LDL, (*P* > 0.05). Among the participants, the prevalence of hypertension, dyslipidemia, diabetes, CAD, stroke, CKD, CLD, and MCCs was 51.4%, 14.5%, 24.2%, 17.7%, 13.4%, 14.0%, 53.0%, and 57%, respectively.

**Table 1:** Basic characteristics of the research object [n (%)]

Characteristics	Total (*N* = 4278)	Male (*n*_1_ = 1861)	Female (*n*_2_ = 2417)	*P*-value
Age				<0.001
65 ∼ 69	1469(34.3)	560(30.1)	909(37.6)	
70 ∼ 74	1472(34.4)	651(35.0)	821(34.0)	
75 ∼ 79	731(17.1)	335(18.0)	396(16.4)	
≥ 80	606(14.2)	315(16.9)	291(12.0)	
Marital status				<0.001
Married/common-law marriage	3379(79.0)	1730(93.0)	1649(68.2)	
Divorce/widowhood/Unmarried	899(21.0)	131(7.0)	768(31.8)	
Education level				<0.001
Primary school and below	1243(29.0)	366(19.7)	877(36.3)	
Junior / High School	1594(37.3)	719(38.6)	875(36.2)	
Secondary school and above	1441(33.7)	776(41.7)	665(27.5)	
Place of domicile				0.238
City	2563(59.9)	1107(59.5)	1456(60.2)	
Town	469(11.0)	221(11.9)	248(10.3)	
Rural district	1246(29.1)	533(28.6)	713(29.5)	
Whether regular night shift				0.001
Yes	920(21.5)	445(23.9)	475(19.7)	
No	3358(78.5)	1416(76.1)	1942(80.3)	
Intensity of exercise				0.336
low intensity	805(18.8)	333(17.9)	472(19.5)	
moderate strength	2838(66.4)	1242(66.7)	1596(66.0)	
high intensity	635(14.8)	286(15.4)	349(14.5)	
Sleep times (hours)				<0.001
< 6	977(22.8)	295(15.9)	682(28.2)	
6 ∼ 8	2879(67.3)	1332(71.5)	1547(64.0)	
> 8	422(9.9)	234(12.6)	188(7.8)	
Smoke status				<0.001
Never	3383(79.1)	988(53.1)	2395(99.1)	
Former	508 (11.9)	496(26.7)	12(0.5)	
Current	387(9.0)	377(20.3)	10(0.4)	
Drinking status				<0.001
Never	3553(83.1)	1249(67.1)	2304(95.3)	
Former	147(3.4)	106(27.2)	41(1.7)	
Current	578(13.5)	506(5.7)	72(3.0)	
Abdominal Obesity				<0.001
Yes	1765(41.3)	551(29.6)	1214(50.2)	
No	2513(58.7)	1310(70.4)	1203(49.8)	
SBP(mmHg)*	133(124, 141)	133(124, 142)	131(124, 141)	0.554
DBP(mmHg)*	75(70,81)	75(70,81)	75(70,81)	0.201
BMI*	24.07(22.42,25.79)	24.07(22.56,25.83)	24.07(22.32,25.73)	0.073
GLU(mmol/L)*	5.22(4.87,5.83)	5.22(4.88,5.87)	5.22(4.86,5.77)	0.059
TC(mmol/l)*	5.14(4.54,5.75)	5.14(4.52,5.68)	5.14(4.57,5.79)	0.068
TG(mmol/L)*	1.32(1.03,1.75)	1.32(1.05,1.75)	1.32(1.03,1.76)	0.651
HDL(mmol/L)*	1.35(1.20, 1.54)	1.35(1.20, 1.54)	1.35(1.21, 1.55)	0.125
LDL(mmol/L)*	2.97(2.51,3.44)	2.97(2.52,3.42)	2.97(2.51,3.45)	0.365
Hypertension				0.074
Yes	2200(51.4)	986(53.0)	1214(50.2)	
No	2078(48.6)	875(47.0)	1203(49.8)	
Dyslipidemia				<0.001
Yes	619(14.5)	209(11.2)	410(17.0)	
No	3659(85.5)	1652(88.8)	2007(83.0)	
Diabetes				0.081
Yes	1034(24.2)	474(25.5)	560(23.2)	
No	3244(75.8)	1387(74.5)	1857(76.8)	
Stroke				<0.001
Yes	574(13.4)	294(15.8)	280(11.60	
No	3704(86.6)	1567(84.2)	2137(88.4)	
CAD				0.705
Yes	757(17.7)	334(17.9)	423(17.5)	
No	3521(82.3)	1527(82.1)	1994(82.5)	
CKD				<0.001
Yes	598(14.0)	331(17.8)	167(11.0)	
No	3680(86.0)	1530(82.2)	2150(89.0)	
CLD				0.452
Yes	2267(53.0)	974(52.3)	1293(53.5)	
No	2011(47.0)	887(47.7)	1124(46.5)	
MCCs				0.645
Yes	2445(57.2)	1071(57.5)	1374(56.8)	
No	1833(42.8)	790(42.5)	1043(43.2)	

*Note* * is skewed distribution, expressed by M (Q1, Q3)

### The basic situation related to the quintile distribution of total dietary branched-chain amino acids among older adults

Table 2 shows the quintile distribution of dietary BCAAs among the participants. Those with high dietary branched-chain amino acid intake (Q5 > 15.721 g/day) were more likely to be male, over 80 years old, and married, with an educational level of secondary school and above. These participants were also more likely to live in the city, participate in high-intensity exercise, have centripetal obesity, be non-drinkers, and have high energy, protein, carbohydrate, fat, and vitamin K intakes rather than low intakes (Q1 < 7.736 g/day).

**Table 2:** The quintile distribution of dietary BCAAs among the participants. [Case (%), (M (P25, P75)]

Characteristics	Q1	Q2	Q3	Q4	Q5
Sex^a^					
Male	279(32.6)	343(40.1)	379(44.3)	412(48.1)	448(52.4)
Female	576(67.4)	513(59.9)	477(55.7)	444(51.9)	407(47.6)
Age^**a**^					
65 ∼ 69	311(36.4)	310(36.2)	289(33.8)	273(31.9)	286(33.5)
70 ∼ 74	324(37.9)	276(32.2)	292(34.1)	286(33.4)	294(34.4)
75 ∼ 79	137(16.0)	153(17.9)	149(17.4)	147(17.2)	145(17.0)
≥ 80	83(9.7)	117(13.7)	126(14.7)	150(17.5)	130(15.1)
Marital status^**a**^					
Married/common-law marriage	627(73.3)	648(75.7)	674(78.7)	697(81.4)	733(85.7)
Divorce/widowhood/Unmarried	228(26.7)	208(24.3)	182(21.3)	159(18.6)	122(14.3)
Education level^**a**^					
Primary school and below	405(47.4)	243(28.4)	255(29.8)	186(21.7)	154(18.0)
Junior / High School	293(34.3)	355(41.5)	299(34.9)	325(38.0)	322(37.7)
Secondary school and above	157(18.4)	258(30.1)	302(35.3)	345(40.3)	379(44.3)
Place of domicile^**a**^					
City	376(44.0)	471(55.0)	522(61.0)	586(68.5)	608(71.1)
Town	126(14.7)	105(12.3)	83(9.7)	82(9.5)	73(8.5)
Rural district	353(41.3)	280(32.7)	251(29.3)	188(22.0)	174(20.4)
Intensity of exercise^**a**^					
low intensity	212(24.8)	163(19.0)	155(18.1)	138(16.1)	137(16.0)
moderate strength	552(64.6)	583(68.1)	572(66.8)	590(68.9)	541(63.3)
high intensity	91(10.6)	110(12.9)	129(15.1)	128(15.0)	177(20.7)
Sleep times (hours)					
< 6	195(22.8)	201(23.5)	209(24.4)	167(19.5)	205(24.0)
6 ∼ 8	561(65.6)	577(67.4)	556(65.0)	607(70.9)	578(67.6)
> 8	99(11.6)	78(9.1)	91(10.6)	82(9.6)	72(8.4)
Smoke status					
Never	698(81.7)	688(80.4)	673(78.6)	649(75.8)	675(78.9)
Former	85(9.9)	87(10.2)	99(11.6)	127(14.8)	110(12.9)
Current	72(8.4)	81(9.4)	84(9.8)	80(9.4)	70(8.2)
Drinking status^**a**^					
Never	741(86.7)	723(84.5)	704(82.2)	705(82.4)	680(79.5)
Former	29(3.4)	25(2.9)	31(3.6)	22(2.6)	40(4.7)
Current	85(9.9)	108(12.6)	121(14.1)	129(15.1)	135(15.8)
Abdominal Obesity^**a**^					
Yes	460(53.8)	504(58.9)	487(56.9)	514(60.0)	548(64.1)
No	395(46.2)	352(41.1)	369(43.1)	342(40.0)	307(35.9)
energy*(kcal/day)^**a**^	919.44(755.05,1169.00)	1203.43(983.28,1412.04)	1319.24(1100.87,1554.40)	1499.04(1262.74,1759.44)	1900.77(1594.05,2259.05)
protein*(g/day)^**a**^	38.44(31.92,42.78)	54.52(50.67,58.35)	68.72(65.14,72.63)	84.98(80.32,89.92)	113.51(102.87,128.42)
carbohydrate*(g/day)^**a**^	264.16(220.80,354.33)	330.26(261.99,409.01)	349.44(280.10,432.96)	376.55(309.40,461.11)	450.74(363.28,528.08)
Fat*(g/day)^**a**^	36.94(29.26,46.64)	50.44(40.89,61.19)	61.98(50.38,73.49)	75.21(61.84,90.50)	99.07(81.63,125.17)

*Note* *was skewed distribution, expressed by M (Q1, Q3).^a^ represents bilateral *P* < 0.05, with statistical significance.Cut off values of BCAAs quartiles are as follows: Q1: Q1<7.736 g/day, Q2: 7.736 g/day ∼ 10.208 g/day, Q3: 10.208 ∼ 12.630 g/day, Q4:12.630 ∼ 15.721 g/day Q5: ≥15.721 g/day

### General demographic characteristics associated with chronic diseases among older adults

Supplementary Table 2 shows that the types of chronic diseases among the participants were related to older age, living in the city, higher educational level, moderate-intensity exercise, night shift, never smoking, higher blood glucose concentration, lower total cholesterol, higher triglyceride concentration, higher isoleucine content, lower leucine content, and lower total branched-chain amino acid concentration. The types of chronic diseases present in the participants were not related to marital status, sleep duration, drinking status, triglyceride, high-density lipoprotein, low-density lipoprotein, and valine content.

### Multivariate logistic regression analysis of isoleucine, leucine, valine and comorbidity of chronic diseases among older adults

Table 3 shows the results of the univariate model and the multivariate model regarding the intakes of the three amino acids and MCCs. Dietary isoleucine was a risk factor for MCCs (OR = 3.388, 95%CI:1.415,8.109) in both the univariate model and the multivariate model with adjustment for confounding factors. In the univariate model, leucine (OR = 0.604, 95%CI:0.430,0.849) was a protective factor for MCCs. However, after adjusting for the covariates, the negative correlation between leucine and MCCs changed and became insignificant. In the gender stratification analysis, after adjusting for covariates such as age, educational level, and BMI, isoleucine (OR = 6.902, 95%CI:1.875,25.402) was a risk factor for MCCs in women, and leucine (OR = 0.506, 95%CI:0.309,0.830) was a protective factor. These associations were not found in men. No significant association between valine and MCCs was observed for both genders.

**Table 3:** Multivariate logistic regression analysis of isoleucine, leucine, valine and comorbidity of chronic diseases among older adults

	Total			Male			Female	
Characteristics	OR	95%CI		OR	95%CI		OR	95%CI
Model 1								
Isoleucine	**5.234**	**2.262** ∼ **12.112**		3.058	0.962 ∼ 9.719		**11.617**	**3.252** ∼ **41.497**
Leucine	**0.604**	**0.430** ∼ **0.849**		0.836	0.516 ∼ 1.356		**0.424**	**0.262** ∼ **0.688**
Valine	0.519	0.261 ∼ 1.034		0.502	0.192 ∼ 1.313		0.442	0.159 ∼ 1.225
Model 2								
Isoleucine	**4.136**	**1.745** ∼ **9.803**		2.299	0.714 ∼ 7.399		**8.075**	**2.228** ∼ **29.268**
Leucine	**0.697**	**0.493** ∼ **0.985**		0.982	0.601 ∼ 1.606		**0.494**	**0.303** ∼ **0.806**
Valine	0.510	0.253 ∼ 1.029		0.502	0.191 ∼ 1.320		0.480	0.172 ∼ 1.340
Model 3								
Isoleucine	**3.388**	**1.415** ∼ **8.109**		1.807	0.549 ∼ 5.950		**6.902**	**1.875** ∼ **25.402**
Leucine	0.726	0.512 ∼ 1.029		1.067	0.646 ∼ 1.763		**0.506**	**0.309** ∼ **0.830**
Valine	0.564	0.278 ∼ 1.146		0.542	0.203 ∼ 1.488		0.526	0.187 ∼ 1.480

*Note* Model 1 did not make any adjustments; model 2 adjusted age and gender; model 3 adjusted age, marital status, education level, smoking, drinking, BMI, TG, TC, and exercise intensity. In the model of gender stratification, gender is not regarded as a covariate

### Dose–response relationship between dietary isoleucine and MCCs

**Fig. 2:**
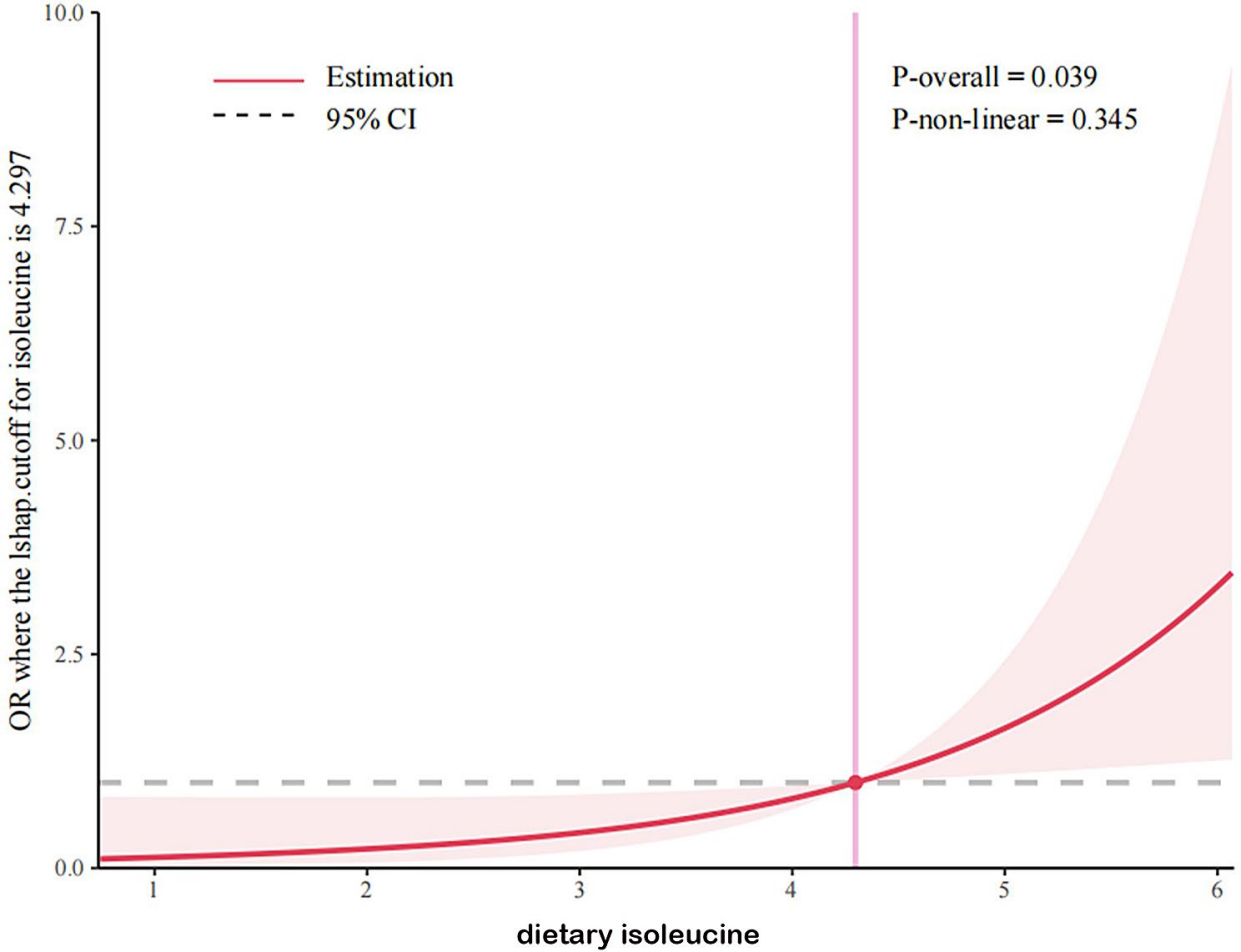
The dose–response relationship between dietary isoleucine and MCCs based on a restricted cubic spline regression model. The figure shows the OR for outcome related to isoleucine after adjusting for age, gender, BMI, marital status, educational level, smoking status, drinking status, leucine, valine, and intensity of exercise. The solid line indicates the OR, and the shadow shape indicates the 95%CI. OR, odds ratio; CI, confidence interval

A restricted cubic spline model was used to fit the dose–response relationship between isoleucine and MCCs. The results showed that when the intake of isoleucine was greater than 4.297 g/d, isoleucine was a risk factor for MCCs, with the risk of MCCs increasing with an increase in intake (see Fig. 2).

### Isoleucine as a risk factor of MCCs based on the ROC curve

**Fig. 3:**
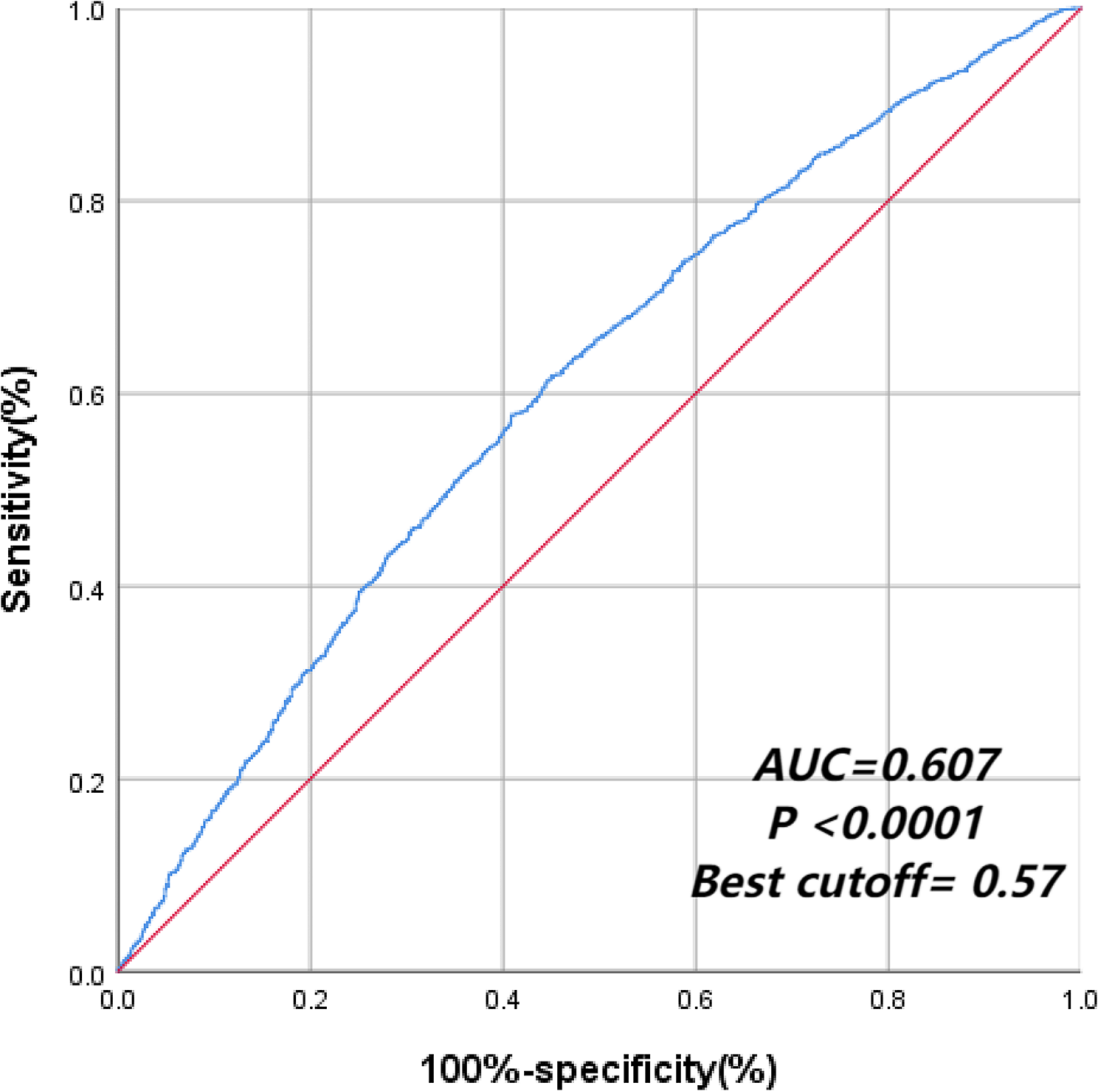
ROC curve for diagnosis of MCCs based on isoleucine

**Fig. 4:**
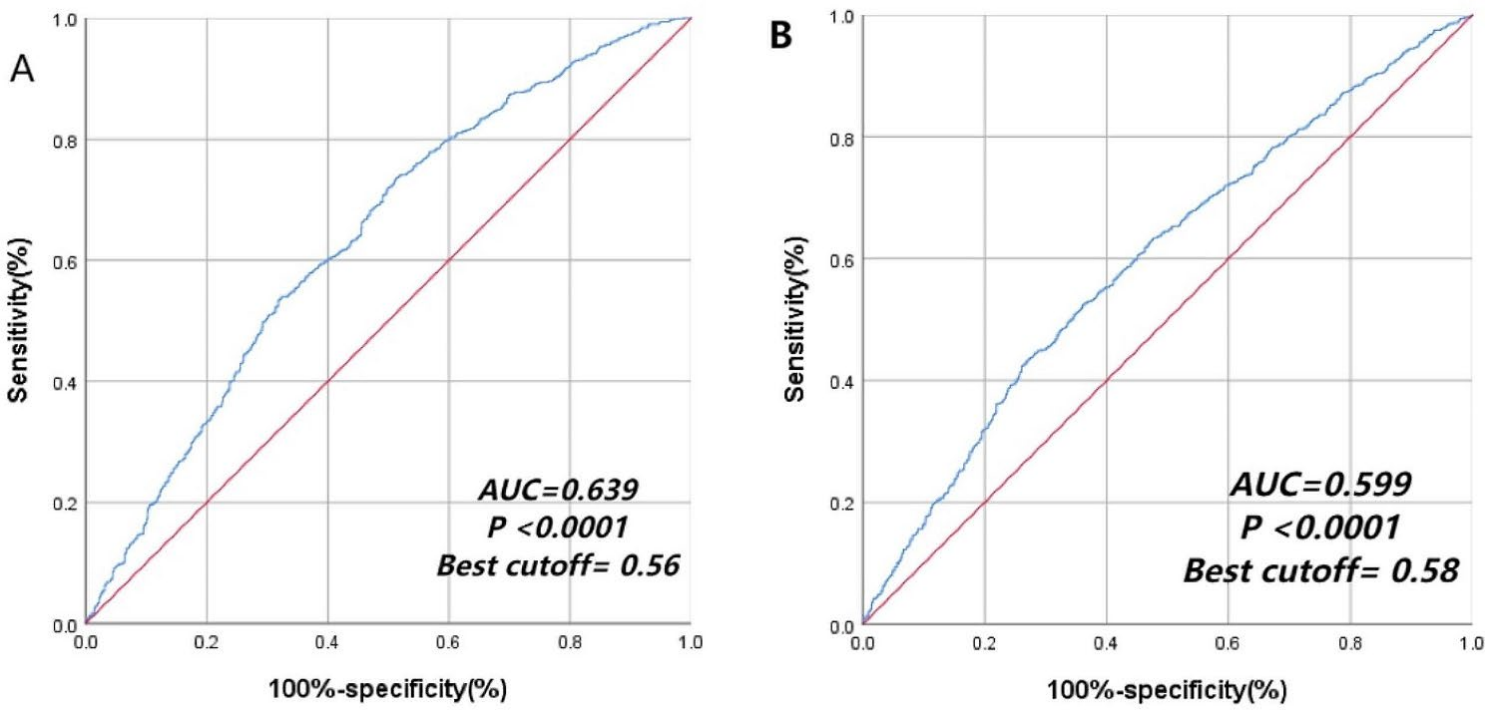
ROC curve for diagnosis of MCCs among males (**A**) and females (**B**) based on isoleucine

The ROC curve shows that the AUC of isoleucine is greater than 0.5, and the identification value of chronic disease comorbidity is shown in Figs. 3 and 4 (*P* < 0.001).

## Discussion

This study explored the association between dietary BCAAs and MCCs (such as hypertension, diabetes, and dyslipidemia) among older adults in Chinese communities. We found that BCAAs (such as isoleucine and leucine) in the diet were significantly associated with MCCs among Chinese older adults. Previous studies have shown that there is a correlation between branched-chain amino acids and multimorbidity among older adults. For example, a Spanish cohort study of 1488 older people over 65 years of age showed that BCAAs, such as isoleucine (OR = 50.3, 95%CI:21.7,78.9) and valine (OR = 15.5, 95%CI:3.10,28.0), were significantly associated with higher multi-disease scores, and isoleucine and valine can be used as risk markers for multimorbidity among older adults [15]. A cross-sectional study of 700 individuals aged ≥ 65 years in Spain found that the ‘amino acid/glycolytic/ketogenic’ factor was associated with a higher incidence of cardiac metabolic diseases [16].Our study did not find an association between valine levels and MCCs, an outcome potentially influenced by a range of factors such as genetic predispositions, ethnic disparities, cultural practices, and individual dietary habits.

After gender stratification, the relationship between dietary isoleucine, leucine, and MCCs was more evident in older women. The presence of estrogen is thought to reduce the risk of cardiovascular disease. After menopause, the incidence and severity of cardiovascular disease (CVD) increase due to a decrease in estrogen levels [33], which may increase the risk of MCCs in women. Leucine is a protective factor for MCCs in older women, possibly because leucine is a receptor for estrogen and has a neuroprotective effec [34], thus reducing the risk of chronic diseases due to changes in estrogen levels.

BCAAs play a key physiological role in aging [35]. Aging reduces mitochondrial biogenesis, stimulates mitochondrial dysfunction, increases oxidative damage, affects biological function, and increases susceptibility to a variety of diseases [36, 37]. The results of the dose–response relationship showed that when the intake of isoleucine was greater than 4.297 g/d, isoleucine might become a risk factor for multiple diseases, and the risk of MCCs increased with an increase in intake. Best cutoff value mean a cutoff value of isoleucine intake which can give higher sensitivity and higher specificity. The AUC value was 0.607, indicating that isoleucine had a certain predictive value for the diagnostic ability of multiple chronic diseases. Therefore, the intake of isoleucine-rich foods, such as grain and grain products, and meat and meat products, should be minimized [38, 39].Older people with a chronic disease can regularly measure the content of isoleucine in the body to prevent MCCs.

Further research is still needed to explore the mechanism underlying the relationship between BCAAs and MCCs. In addition, our study found that there was a significant correlation between dietary BCAAs and age, gender, marital status, educational level, household registration, and exercise intensity, among other factors. This finding suggests that these factors may play a role in the regulation of the association between BCAAs and MCCs. Future research needs to consider the interaction of these factors to better understand the mechanism underlying the association between BCAAs and MCCs.

Although our results provide important clues about the association between dietary BCAAs and MCCs among older adults in Chinese communities, there are some limitations. First, because this study adopted a cross-sectional survey design, it was impossible to determine the causal relationship. Therefore, long-term follow-up studies are needed to verify our findings. Second, this study only examined the association between BCAAs and MCCs, and the relationship between other essential amino acids and MCCs was not investigated. In future research, it is necessary to further explore the association of dietary amino acids with MCCs. Finally, this study only involved older adults living in Nanshan District, Shenzhen City, China. Due to many factors such as economy and region, the generalization of the results needs to be carefully evaluated.

In summary, our results **suggest** that there may be a significant association between dietary BCAAs and MCCs among older adults in Chinese communities; in particular, isoleucine may be a risk factor for MCCs among older adults. This finding provides a basis for further research on the mechanism underlying the relationship between BCAAs and MCCs, which will provide new perspectives and strategies for the prevention and treatment of MCCs. Future research should also consider the moderating role of other factors and use a long-term follow-up study design to verify these findings.

## Conlusion

This study preliminarily explored the relationship between dietary branched-chain amino acids and MCCs among older adults, showing that isoleucine may be a risk factor for MCCs. However, the causal relationship between isoleucine and MCCs was not studied. Future studies should recruit a larger sample size and use a long-term follow-up study design to verify these findings, which will provide new strategies for the prevention and treatment of MCCs.

### Electronic supplementary material

Below is the link to the electronic supplementary material.

Supplementary Material 1

## Data Availability

No datasets were generated or analysed during the current study.
