# Supplementary material for: Association between dietary branched-chain amino acids and multiple chronic conditions among older adults in Chinese communities
Source: Nutr Metab (Lond). 2024 Jul 30;21:56. doi: 10.1186/s12986-024-00825-9 (PMC11290232; doi:10.1186/s12986-024-00825-9)
Supplement: Supplementary file 1 — Supplementary Material 1 [file 12986_2024_825_MOESM1_ESM.docx]

**Table S1.** English acronym list.

| **clipped word** | **English full name** |
| --- | --- |
| BMI | Body Mass Index |
| GLU | Blood Glucose |
| HDL-C | High Density Lipoprotein Cholesterol |
| LDL-C | Low Density Lipoprotein Cholesterol |
| TC | Total Cholesterol |
| TG | Triglyceride |
| BP | blood pressure |
| SBP | systolic blood pressure |
| DBP | diastolic blood pressure |
| BCAA | branched chain amino acid |

**Table S2.** General demographic characteristics of chronic diseases among older adults (n = 4435). [ Example ( % ), ( M ( P25, P75 ) ]

| **Characteristics** | **Number** | **Types of chronic diseases** | | | | ***P-value*** |
| --- | --- | --- | --- | --- | --- | --- |
|  |  | 0 | 1 | 2 | 3 or more |  |
| Sex |  |  |  |  |  | 0.123 |
| Male | 1861 | 232(47.5) | 412(43.1) | 449(41.2) | 768(44.0) |  |
| Female | 2417 | 256(52.5) | 544(56.9) | 640(58.8) | 977(56.0) |  |
| Age |  |  |  |  |  | ＜0.001 |
| 65~69 | 1469 | 224(45.9) | 390(40.8) | 378(34.7) | 477(27.3) |  |
| 70~74 | 1472 | 164(33.6) | 325(34.0) | 392(36.0) | 591(33.9) |  |
| 75~79 | 731 | 62(12.7) | 146(15.3) | 171(15.7) | 352(20.2) |  |
| ≥80 | 606 | 38(7.8) | 95(9.9) | 148(13.6) | 325(18.6) |  |
| Place of domicile |  |  |  |  |  | ＜0.001 |
| City | 2563 | 240(49.2) | 539(56.4) | 634(58.2) | 1150(65.9) |  |
| Town | 469 | 56(11.5) | 105(11.0) | 127(11.7) | 181(10.4) |  |
| Rural district | 1246 | 192(39.3) | 312(32.6) | 328(30.1) | 414(23.7) |  |
| Marital status |  |  |  |  |  | 0.833 |
| Married/common-law marriage | 3379 | 393(80.5) | 756(79.1) | 856(78.6) | 1374(78.7) |  |
| Divorce/widowhood/Unmarried | 899 | 95(19.5) | 200(20.9) | 233(21.4) | 371(21.3) |  |
| Education level |  |  |  |  |  | ＜0.001 |
| Primary school and below | 1243 | 177(36.3) | 311(32.5) | 338(31.0) | 417(23.9) |  |
| Junior / High School | 1594 | 179(36.7) | 358(37.4) | 423(38.8) | 634(36.3) |  |
| Secondary school and above | 1441 | 132(27.0) | 287(30.1) | 328(30.2) | 694(39.8) |  |
| Whether regular night shift |  |  |  |  |  | 0.007 |
| No | 3358 | 406(83.2) | 764(79.9) | 855(78.5) | 1333(76.4) |  |
| Yes | 920 | 82(16.8) | 192(20.1) | 234(21.5) | 412(23.6) |  |
| Intensity of exercise |  |  |  |  |  | 0.021 |
| Low intensity | 805 | 83(17.0) | 181(18.9) | 218(20.0) | 323(18.5) |  |
| Moderate strength | 2838 | 312(63.9) | 622(65.1) | 708(65.0) | 1196(68.5) |  |
| High intensity | 635 | 93(19.1) | 153(16.0) | 163(15.0) | 226(13.0) |  |
| Sleep times ( hours ) |  |  |  |  |  | 0.828 |
| <6 | 977 | 102(20.9) | 218(22.8) | 248(22.8) | 409(23.4) |  |
| >8 | 422 | 49(10.0) | 96(10.0) | 98(9.0) | 179(10.3) |  |
| Smoke status |  |  |  |  |  | ＜0.001 |
| Never | 3383 | 364(74.6) | 761(79.6) | 877(80.6) | 1381(79.1) |  |
| Former | 508 | 54(11.1) | 92(9.6) | 130(11.9) | 232(13.3) |  |
| Current | 387 | 70(14.3) | 103(10.8) | 82(7.5) | 132(7.6) |  |
| Drinking status |  |  |  |  |  | ＜0.001 |
| Never | 3552 | 396(81.1) | 790(82.7) | 905(83.1) | 1461(83.7) |  |
| Former | 147 | 40(8.2) | 46(4.8) | 30(2.8) | 31(1.8) |  |
| Current | 578 | 52(10.7) | 119(12.5) | 154(14.1) | 253(14.5) |  |
| BMI* |  | 24.07(22.17,25.75) | 24.07(22.37,25.78) | 24.07(22.55,25.81) | 24.07(22.42,25.80) | 0.748 |
| SBP* |  | 133(124,141) | 133(125,141) | 133(124,142) | 133(124,141) | 0.869 |
| DBP* |  | 75(70,81） | 75(70,81) | 75(70,80) | 75(70,81) | 0.840 |
| GLU* |  | 5.20(4.81,5.60) | 5.22(4.85,5.75) | 5.22(4.87,5.85) | 5.22(4.90,5.90) | 0.028 |
| TC* |  | 5.14(4.60,5.86) | 5.14(4.60,5.74) | 5.14(4.61,5.79) | 5.14(4.48,5.67) | 0.016 |
| TG* |  | 1.32(1.07,1.82) | 1.32(1.03,1.74) | 1.32(1.03,1.78) | 1.32(1.03,1.73) | 0.846 |
| HDL-C* |  | 1.35(1.20,1.53) | 1.35(1.20,1.55) | 1.35(1.20,1.56) | 1.35(1.21,1.53) | 0.653 |
| LDL-C* |  | 2.97(2.58,3.49) | 2.97(2.57,3.46) | 2.97(2.52,3.47) | 2.97(2.46,3.40) | 0.039 |

Note : * is skewed distribution, expressed by M ( Q1, Q3 ).
